# SP1 lactylation promotes endometrial cancer cell stemness via regulation of CENPL expression

**DOI:** 10.1016/j.gendis.2025.101962

**Published:** 2025-12-05

**Authors:** Jian Ma, Ziyu Zhang, Bo Wang, Wantong Wang, Fanfei Kong, Xiao-Xin Ma

**Affiliations:** Department of Obstetrics and Gynecology, Shengjing Hospital of China Medical University, Shenyang, Liaoning 110004, China

Cancer stem cells (CSCs) are resistant to anti-tumour therapies and are associated with metastasis and recurrence. High concentrations of lactic acid can promote the maintenance of tumour cell stemness.^1^ We previously reported that SOX2 promotes the malignant phenotype and stemness of ECCs and ECSC.^2^ SP1 is a transcription factor. It promotes the proliferation and invasion of endometrial cancer (EC) cells.^3^ In lung adenocarcinoma, SP1 promotes stemness by modulating the expression of key stemness genes, including OCT4, Nanog, and CTGF.^4^ Immunotherapy represents a potential treatment option for patients with advanced EC. However, only a limited number of patients benefit from the currently available regimens. Studies have shown that the TGF-β/PD-L1 bispecific antibody can enhance anti-tumor immunity,^5^ which provides new insights for the treatment of endometrial cancer.

We collected 26 stem cell genomes representing self-regeneration characteristics and obtained the sample score of the gene set through ssGSEA. Based on the consensus score of the CDF curve, *k* = 3 was the optimal value. In addition, PCA showed that the ssGSEA scores based on 26 stem cell gene sets could be used to divide the data into three subtypes: SCE_H, SCE_M, and SCE_L (Fig. S1A–S1D). Estimation of the immune infiltration level of the dataset with the estimate algorithm revealed the following results: Immunocore sort (SCE_L > SCE_M > SCE_H), stromal score (SCE_H > SCE_L > SCE_M), and tumour purity sort (SCE_M > SCE_H > SCE_L) (Fig. S1E and S1F).

To identify the characteristic marker genes of EC stem cell subtypes, we used the expression data of 3694 differential genes as input files and weighted gene co-expression network analysis (WGCNA) to describe the association pattern within the genes. Subsequently, 3694 differential genes were divided into 11 modules, of which yellow and turquoise had the strongest correlation with stem cell subtypes (Fig. S2A). To identify genes with prognostic value in stem cell subtypes, we analysed 143 genes in the turquoise SCE_M module (Table S2). Univariate Cox survival analysis was then performed on the EC-TCGA dataset, and the 143 genes were screened based on the relationship between their expression and patient prognosis (*p* < 0.01). We searched an online database assistant for clinical bioinformatics (https://www.aclbi.com/static/index.html#/) to analyze the prognostic impact of batch screening of 143 genes in endometrial cancer patients. A total of 9 genes significantly associated with prognosis were obtained, *ACOXL, C1orf112, CDC25A,* and *CENPL* (Fig. S2B). Next, we performed Spearman's rank correlation to assess the relationship between the nine stem cell-related genes and common stem cell markers: ALDH1A1, NANOG, POU5F1, CD133, and SOX2 (Fig. S2C). To evaluate the expression of *ACOXL, C1orf112, CDC25A,* and *CENPL* in 16 normal endometrial and 27 EC tissues, RT-PCR was performed. *ACOXL, C1orf112, CDC25A,* and *CENPL* expression was up-regulated in EC tissues compared to normal tissue samples (Fig. S2D).

The results of immunohistochemistry and Western blotting showed that the expression level of CENPL was higher in endometrial carcinoma tissues than in normal endometrial tissues (Fig. 1A; Fig. S3A and S3B). To investigate the carcinogenic effect of CENPL in EC, we knocked it down in Ishikawa cells. *CENPL* knockdown inhibited Ishikawa cell proliferation, invasion and spheroid-formation (Fig. 1B–D). In order to explore the binding ability between the SOX2 and CENPL proteins, AlphaFold3 was used to predict the SOX2-CENPL complex. The results showed that the surface matching between the two was good, which was conducive to the formation of stable binding interactions (Fig. S3C). Further molecular dynamics simulations were used to analyze the interaction between the SOX2 protein and CENPL. The RMSD and RMSF curves indicate that SOX2 and CENPL proteins have strong affinity and can form stable complex structures, thereby exerting their biological functions (Fig. S3D–S3J). Co-IP further suggested the interaction between CENPL and SOX2 (Fig. S3K). Immunofluorescence co-localization results showed that CENPL and SOX2 were co-located in the nucleus (Fig. 1E). Western blotting showed that knockdown of CENPL decreased the expression of SOX2 (Fig. 1F). Moreover, overexpression of SOX2 promoted lactate production (Fig. 1G). Rescue experiments indicated that the malignant inhibitory effects of CENPL silencing on the proliferation, invasion and spheroid-formation of Ishikawa cells were significantly reversed by SOX2 overexpression (Fig. 1H–J). The mass spectrometry results further indicated that K19 of SP1 serves as a lactylation site (Fig. 1K). IP and Western blotting assays showed that a glycolysis inhibitor (2-DG) significantly reduced the level of SP1 lactate modification and the expression of SP1 (Fig. 1L). The transfection of Ishikawa cells with mutant SP1 (K19R) caused a significant decrease in lactate modification levels compared with cells transfected with wild-type SP1 (WT; Fig. 1M). Additionally, the mutant SP1 (K19R)-transfected Ishikawa cells exhibited reduced proliferation, invasion spheroid-formation (Fig. 1N–P). We next determined the function of the mutant SP1 (K19R) in an *in vivo* tumor model. In the experiment of nude mice, the results showed that the mutant SP1 (K19R) transfection decreased the tumor volume compared with the control group (Fig. 1Q). Immunohistochemical analysis showed that the mutant SP1 (K19R) group had lower expression of SOX2 than the control group (Fig. 1R).

**Figure 1:**
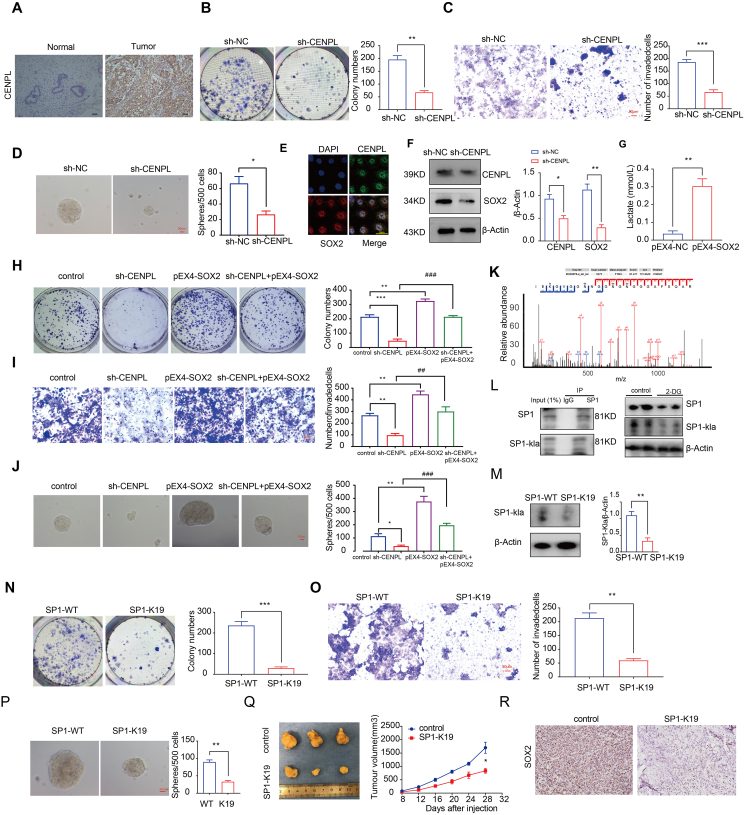
SP1 lactylation promotes the stemness of endometrial cancer cells. **(A)** The protein expression of CENPL in 30 EC tissues and 20 normal tissues was determined by immunohistochemistry. **(B)** The effect of CENPL expression on the proliferation of Ishikawa cells detected using colony formation assay. Data are presented as mean ± SEM (*n* = 3 per group); ∗*p* < 0.05, ∗∗*p* < 0.01 compared with the NC group. **(C)** Transwell analysis was used to determine the number of invading cells. Data are presented as mean ± SEM (*n* = 3 per group); ∗*p* < 0.05, ∗∗*p* < 0.01, ∗∗∗*p* < 0.001 compared with the NC group. **(D)** Stem cell evaluation of Ishikawa cells using a spheroid-formation assay. Data are presented as mean ± SEM (*n* = 3 per group); ∗*p* < 0.05, ∗∗*p* < 0.01 compared with the NC group. **(E)** Immunofluorescence experiment. **(F)** Western blot analysis of SOX2 abundance in response to CENPL in Ishikawa cells. Data are presented as mean ± SEM (*n* = 3 per group); ∗*p* < 0.05, ∗∗*p* < 0.01 compared with the NC group. **(G)** Lactate production by Ishikawa cells. Data are presented as mean ± SEM (*n* = 3 per group); ∗*p* < 0.05, ∗∗*p* < 0.01, compared with the NC group. **(H)** Ishikawa cell proliferation. **(I)** invasion. **(J)** spheroid-formation. **(H**–**J)** Data are presented as mean ± SEM (*n* = 3 per group); ∗*p* < 0.05, ∗∗*p* < 0.01, ∗∗∗*p* < 0.001 compared with the NC group, ^#^*p* < 0.05, ^##^*p* < 0.01, ^###^*p* < 0.001 compared with the sh-CENPL group **(K)** Collision-induced dissociation (CID) analysis to identify modification sites. (**L)** Quantification of pan-Kla and SP1 expression in Ishikawa cells treated with 2-DG (20 mM) for 24 h (*n* = 3 per group). **(M)** SP1 lactylation and SP1 expression in Ishikawa cells. Data are presented as mean ± SEM (*n* = 3 per group); ∗*p* < 0.05, ∗∗*p* < 0.01, ∗∗∗*p* < 0.001 compared with the WT group. **(N–P)** Ishikawa cell proliferation (O) invasion, and (P) spheroid-formation, Data are presented as mean ± SEM (*n* = 3 per group) ∗*p* < 0.05, ∗∗*p* < 0.01, ∗∗∗*p* < 0.001 compared with the WT group. **(Q)** Nude mice bearing tumors and a specimen from each respective group are shown (*n* = 3 per group). **(R)** Protein expression of SOX2 in nude mouse xenograft tissues; positive cells are stained brown (*n* = 3 per group).

The ribbon diagram of the human SP1 protein crystal structure suggests that the K19 site is the lactylation site. The tertiary structure showed that K19 is located in the inhibitory domain of the SP1 protein (Fig. S4A). According to the JASPAR database, we predicted that SP1 has binding sites in the promoter region of *CENPL* and may serve as a transcription factor to target and regulate the cancer-promoting effect of CENPL. Accordingly, we examined the relationship between SP1 and CENPL. The ChIP results showed that SP1 could bind to the promoter region of *CENPL* (Fig. S4B and S4C). Meanwhile, following mutation of the SP1 lactylation site, the expression level of *CENPL* mRNA was reduced, and the abundance of CENPL and SOX2 was reduced (Fig. S4D and S4E). To identify potential inhibitors, we performed virtual screening campaigns against two targets: CENPL and the K19 lactylation site of SP1. For CENPL, energy-based scoring and subsequent standard precision (SP) docking yielded 185 primary hits (Table S3). Analysis of the predicted binding poses led to the selection of five promising candidates (Fig. S4F and S4G). A similar screening against the SP1 lactylation site identified 100 top-scoring compounds (Table S4). From this set, six lead compounds were prioritized based on their stable predicted binding modes and favorable binding energies (Fig. S4H and S4I). The above compounds stably bind to SP1 lactylation sites or CENPL and may be potential therapeutic drugs for EC. Tumor mutation burden (TMB) and microsatellite instability (MSI) are important biomarkers for predicting the efficacy of immunotherapy in endometrial cancer. The results showed that the expression of SP1 and CENPL was significantly positively correlated with MSI in the TCGA database, providing a theoretical basis for developing potential EC drugs targeting the CENPL/SP1 axis (Fig. S4J). A working model is presented (Fig. S4H).

Our results suggest that the EC stem cell subtypes are involved. SP1 lactosylation then regulates the stemness phenotype of EC cells by regulating *CENPL* transcription. *In vitro* CENPL knockdown effectively inhibits Ishikawa cell clonogenesis, invasion, and stemness. CENPL promotes the stemness of EC cells by interacting with SOX2. Our findings suggest potential therapeutic targets for EC and can help to identify potentially effective EC drugs.

## CRediT authorship contribution statement

**Jian Ma:** Writing – original draft. **Ziyu Zhang:** Formal analysis. **Bo Wang:** Validation. **Wantong Wang:** Validation. **Fanfei Kong:** Validation. **Xiao-Xin Ma:** Writing – review & editing.

## Ethics approval and consent to participate

The study protocol was reviewed and approved by the Scientific Research and New Technology Ethical Committee of the Shengjing Hospital of China Medical University. Ethical number: Human: 2018PS251K; Animal: 2018PS136K.

## Availability of data and materials

The datasets and code used during the current study are available from the corresponding author upon reasonable request.

## Funding

This work was supported by the 10.13039/501100001809National Natural Science Foundation of China (No. 82203469) and Leading Talents of Innovation in Liaoning Province, China (No. XLYC1902003).

## Conflict of interests

The authors declare that they have no competing interests.
