# Supplementary material for: SP1 lactylation promotes endometrial cancer cell stemness via regulation of CENPL expression
Source: Genes Dis. 2025 Dec 5;13(4):101962. doi: 10.1016/j.gendis.2025.101962 (PMC12989826; doi:10.1016/j.gendis.2025.101962)
Supplement: Multimedia component 1 [file mmc1.docx]

**Supplementary Data**

**Materials and methods**

**Stem cell signature collection**

In this analysis, the 26 gene sets associated with human stem cells were sourced from the StemChecker platform (http://stemchecker.sysbiolab.eu/). The gene sets were derived through computational methods involving expression validations, RNA interference screenings, and comprehensive literature reviews.

**Data processing**

From TCGA database (https://portal.gdc.cancer.gov/repository), we downloaded EC RNA-seq data, VarScan mutation data, and clinical information for 35 normal and 552 EC samples. The ensemble database was subsequently used for gene annotation.

**Identification of EC subtypes based on stem cell gene sets**

We conducted single sample gene set enrichment analysis (ssGSEA) to measure the enrichment levels of the 26 stem cell gene sets across individual EC samples. The ConsensusClusterPlus package within the R software was utilized for the consensus clustering of genes and the categorization of stem cell subtypes. A maximum of six clusters was established, and the clustering process was executed over 50 iterations, employing 80% of the samples on each occasion, with the parameters set to clusteralg = ‘HC’ and innerlinkage = ‘ward D2’. Cluster heat maps were generated and analyzed using the pheatmap package (version 1.0.12) in R. The ConcensusClusterPlus software package to divide all tumour samples into K (k = 2–10) different subtypes. The optimal number of clusters was identified based on the cluster score obtained from cumulative distribution function (CDF) and delta area curves. Additionally, principal component analysis (PCA) was applied to reduce dimensionality, thereby confirming the consistency of the consensus clusters.

**Evaluation of immune cell infiltration, tumour purity, and matrix content in EC**

Transcriptome data were used to infer the composition and infiltration of tumour cells through the estimate algorithm, and identify characteristic immune cells related to stromal cell infiltration. The estimate algorithm was implemented using the R software. We established the number of permutations at 1,000 and employed a significance threshold of p < 0.05 for screening purposes. The differences among the molecular subtypes of each stem cell within EC were assessed using the Kruskal–Wallis test.

**Assessment of immune cell subsets across various molecular subtypes of EC**

The CIBERSORT package in R was utilized to analyze the variations in the infiltration levels of 22 immune cell types across different molecular subtypes of EC stem cells.

**Survival analysis**

Kaplan–Meier curves were constructed to assess the variations in survival rates among patients with EC across different datasets of stem cell subtypes. Comparisons were made regarding overall survival (OS), relapse-free survival (RFS), progression-free survival (PFS), and disease-specific survival (DSS) among the various groups. The Log-rank test was applied, with a significance level established at *P*< 0.05.

**Chemical response prediction**

Utilizing the FPKM RNA-seq expression profiles from TCGA, we aimed to forecast chemotherapy effectiveness in each group based on data from the pharmacogenomics database (Genomics of Drug Sensitivity in Cancer (GDSC), https://www.cancerrxgene.org/). This prediction was conducted with the R software package ‘pprophetic’, which estimated the half-maximum inhibitory concentration (IC50) value for each sample using ridge regression. Additionally, the prediction’s accuracy was assessed through 10-fold cross-validation, in accordance with the GDSC training set.

**Gene co-expression network analysis**

Using the limma package in R, we identified a total of 3,694 differentially expressed genes (DEGs) between the EC and normal groups, under the stringent thresholds of |log2FC| > 1 and adjusted p-value < 0.05. We conducted a weighted gene co-expression network analysis (WGCNA) on the 3,694 DEGs. Network construction and module detection were based on the unsigned topological overlap matrix, using an optimal soft-threshold power of 6, a minimum module size of 30 genes, and a branch merge cut height of 0.25. To identify the key gene modules associated with EC stem cell subtypes, we calculated the Pearson correlations between module eigengenes and the subtypes.

**TMB/MSI correlation analysis**

Downloaded STAR-counts data and corresponding clinical information for EC tumors from the TCGA database (https://portal.gdc.cancer.gov). Extracted data in TPM format and performed normalization using the log2(TPM+1) transformation. Spearman’s correlation analysis used to describe the correlation between quantitative variables that do not follow a normal distribution. Spearman correlation analysis between TMB/MSI and gene expression.

**Cell culture**

Ishikawa cells were grown in RPMI 1640 medium supplied by Gibco (Carlsbad, CA, USA). These cell lines were sourced from the Institute of Biochemistry and Cell Biology at the Chinese Academy of Sciences, located in Shanghai, China.

**Transfection of Ishikawa cells**

Cells were transfected with shRNAs against CENPL (sh-CENPL) and their negative control (NC) lentivirus were obtained from Genechem (GenePharma, Shanghai, China) The sh-CENPL sequence was 5′-UAUUUGACAGAACUGGCAATT-3′. Construction of SP1 wild-type (WT) and SP1 mutant (K19R) lentivirus (Syngen tech, Beijing, China) Overexpression SOX2 plasmids were purchased from GenePharma (Shanghai, China). Using Lipofectamine™ 3000 (Thermo Fischer Scientific, Carlsbad, CA, USA) as per the manufacturer’s instructions.

**Cell invasion assay**

Following the sterilization of the Transwell chamber, Matrigel (with an 8 μm pore size; Corning, NY, USA) was applied and incubated overnight at 37 °C to allow the gel to set. Cells in the logarithmic growth phase were collected, centrifuged, and the supernatant was removed. The cells were then resuspended in a serum-free medium. Next, 800 μL of culture medium containing 10% fetal bovine serum was added to a separate 24-well plate, into which the Transwell chamber was subsequently placed. A cell suspension of 5 × 10^4 cells was introduced into the upper chamber and incubated for 24 hours at 37 °C with 5% CO2. After incubation, the Transwell chamber was taken out, and any non-adherent cells in each well were discarded along with the medium in the upper chamber. The wells were air-dried and then fixed with 4% paraformaldehyde. Crystal violet staining solution was applied, followed by rinsing the cells with phosphate-buffered saline (PBS). Images were captured using a microscope, and the cell count was recorded.

**Clonogenic assay for cell proliferation**

In each experimental group, Ishikawa cells were initially plated at a density of 500 cells per well in a 6-well plate. Following a two-week incubation period, the culture medium was discarded, and the cells were rinsed with PBS. Next, they were fixed using 4% paraformaldehyde for 20 minutes, followed by a 5-minute staining process with 0.2% crystal violet. After thorough rinsing with distilled water, the cells were allowed to dry. A microscopy examination was conducted to capture images of the cells. The cloning efficiency was determined using the formula: (number of cell clones/total number of cells) × 100%.

**Tumour spheroidisation detection**

Ishikawa cells from each experimental group were cultured in DMEM/F12 medium supplemented with 20 ng/mL EGF, 20 ng/mL bFGF, and 2% B27. The cells were created as a single-cell suspension and then distributed into a low-adherence 96-well plate, where each well received 100 μL of spheroid-forming medium along with 10 cells. Following a two-week incubation period, the diameters of the spheroids were assessed using a microscope, and the number of spheroids formed per 100 cells was calculated.

**RNA extraction and quantitative RT-PCR**

Total RNA was extracted from cultured cells and tissues using TRIzol reagent (Takara, Shiga, Japan). For the real-time PCR analysis, this total RNA underwent reverse transcription to cDNA via the PrimeScript™ RT-PCR Kit (Takara) and was subsequently amplified using SYBR® TB Green™ Premix Ex Taq II (Takara). The specific PCR primers were developed by Sangon Biotech Co., Ltd. (Shanghai, China). The expression fold change was determined using the 2^-∆∆Ct^ method, employing GAPDH as the internal reference. The primer sequences are listed in Additional file Table S1.

**Immunohistochemistry**

Lactylation was assessed through immunohistochemistry (Jingjie PTM BioLabs, Hangzhou, China). The evaluation of positive expression depended on whether the cytoplasmic staining exhibited a brownish-yellow or brown hue. Samples were rated with a scoring system: 0 for no staining, 1 for light yellow, 2 for yellow, and 3 for brown or sepia. Additionally, scores were allocated based on the proportion of positively stained cells, with a count of negative cells marked as 0. The percentage of positive cells was categorized as follows: less than 10% was scored as 1, 10% to 50% as 2, 50% to 75% as 3, and 75% or more as 4. The total score was derived from the multiplication of these two scores, and the results were classified accordingly: ≤ 2, negative; 3–4, weak positive (+); 5–8, medium positive (++); 9–12, strong positive (+++). Low expression was indicated by -/+, and high expression was indicated by ++/+++. The assessment of the results was conducted by two senior pathologists who were unaware of the patient information. Each sample was examined independently to quantify the positive cell count and assess the background. If any discrepancies arose, a third pathologist was brought in to make a definitive judgment.

**Immunofluorescence**

Cells were fixed in 4% paraformaldehyde for 15 minutes and then permeabilized using 0.5% Triton-X-100 for 20 minutes. After washing with PBS, the cells were blocked with serum for 1.5 hours. Subsequently, they were incubated with primary antibodies at 4 °C for 16 hours (all primary antibodies utilized in this research are listed in Additional file Table S1). The following day, the cells were treated with a fluorescent-dye conjugated secondary antibody and stained with DAPI for 5 minutes. Images were acquired using an inverted fluorescent microscope at 20× magnification for analysis.

Determination of lactic acid content

The lactate levels in the cells were measured using a lactic acid (LD) assay kit (Jiancheng Bioengineering Institute, Nanjing, China), following the guidelines provided by the manufacturer**.**

Western blotting

Total proteins were isolated using a total protein extraction kit (Beyotime Biotechnology, Shanghai, China), while nuclear proteins were extracted via a nuclear protein extraction kit from the same manufacturer, both following the provided protocols. For the electrophoretic separation of proteins, a 10% sodium dodecyl sulfate (SDS)-polyacrylamide gel was utilized, and the proteins were subsequently transferred onto a polyvinylidene fluoride (PVDF) membrane (Millipore, Massachusetts, USA). The membrane was then blocked with skim milk and incubated with primary antibodies at the concentrations suggested by the manufacturer for 16 hours at 4 °C. (All primary antibodies used in this study are listed in Additional file Table S1). Subsequently, the membrane was probed with an appropriate secondary antibody for 1.5 h at 25 °C. After repeated washing, the film was developed using the Quantum One imaging software (Bio-Rad, California, USA). The intensity of the protein bands was quantified using ImageJ v.1.48.

**LC‒MS/MS analysis and database search**

LC‒MS/MS analysis was performed by Jingjie PTM BioLabs (Hangzhou, China).

**Chromatin Immunoprecipitation (ChIP)**

The ChIP assay was conducted following the manufacturer’s instructions using the ChIP kit from Active Motif (Shanghai, China). Briefly, the procedure involved cross-linking the cells and ultrasonically fragmenting the chromatin. Subsequently, the chromatin solution was treated with anti-SP1 antibody (CST), anti-RNA polymerase II (as a positive control), or normal mouse IgG (as a negative control) and incubated overnight at 4 °C. Afterward, the protein and DNA were de-crosslinked, allowing for DNA purification and enrichment. Primers were designed and synthesized based on the predicted binding sites within the promoter region. qRT-PCR was performed with the primers. The primer sequences are listed in Additional file Table S1.

**AlphaFold3 Predicted**

The SOX2–CENPL protein complex was predicted using AlphaFold3 based on their amino acid sequences (https://alphafoldserver.com/). The resulting structure was prepared in Schrödinger 2019-1 using the Protein Preparation Wizard, during which missing hydrogen atoms were added and incomplete bond information was corrected. Energy minimization was carried out with the OPLS3e force field in a water solvent model to relieve steric clashes, employing sequential steepest descent and conjugate gradient algorithms (maximum 5000 iterations each). The geometry was further refined under OPLS3e constraints. Binding affinity was estimated using the MM-GBSA module with the Minimize sampling method and the VSGB solvation model. Final structural visualization and analysis were performed in PyMOL 2.1.

**Molecular Dynamic Simulation**

To investigate the binding mechanism between SOX2 and CENPL, molecular dynamics (MD) simulations were performed on the screened protein–protein complexes using GROMACS 2020 with the AMBER99SB-ILDN force field and the TIP3P water model. The complexes were solvated in a cubic box with a minimum distance of 1.0 nm between any protein atom and the box edge. System neutrality was achieved by adding Na⁺or Cl⁻counterions based on docking results.

The MD workflow comprised four stages: energy minimization, heating, equilibration, and production simulation. Initially, heavy atoms of the proteins (and small molecules, if present) were constrained, and water molecules were energy-minimized for 10,000 steps (5,000 steps of steepest descent followed by 5,000 steps of conjugate gradient). The constraints were then released, and the entire system underwent another 10,000-step minimization using the same protocol.

Subsequently, the system was gradually heated from 0 to 300 K over 50 ps, followed by equilibration under the NPT ensemble for 50 ps. The production MD simulation was then run for 100 ns under the NPT ensemble, with trajectory snapshots saved every 5 ps. Post-simulation analyses were conducted using the trjconv module, and ligand–protein binding free energies were calculated via the gmx_MMPBSA method within GROMACS 2020.

**Protein molecular docking**

Protein–protein docking was performed using the HDOCK server, which applies a hybrid docking strategy treating both proteins as rigid bodies and sampling the entire surface as the potential binding interface. For each pair, 100 docking conformations were generated, and docking scores were calculated using the ITScorePP iterative scoring function, where more negative values indicate higher model compatibility. The top-scoring conformation was selected and further refined in MOE 2019.1 to resolve potential steric clashes arising from rigid docking. Energy minimization was conducted using the Amber10:ETH force field with an explicit water solvation model, employing a two-step protocol of steepest descent followed by conjugate gradient (maximum 5,000 iterations). The optimized complexes were visualized and analyzed with PyMOL 2.1.

**Virtual screening process**

The crystal structure of the target protein was obtained from the protein database (PDB database, www.rcsb.org). Next, we used the Protein Preparation Wizard module to hydrogenate the protein, remove water molecules, SAM, ethylene glycol, and SEP, repair missing residues, and add side chains. The LigPrep Module was used to perform hydrogenation, energy optimization, and to construct the 3D structure. The compound library for this docking was procured primarily from the Selleck compound entity library (4648) https://www.selleck.cn/screening/inhibitor-library.html). The LigPre module of the Maestro11.9 platform was employed to protonation and minimize the energy of all compounds; OPLS3e was selected as the force field. The virtual screening was performed using the Schrödinger Maestro (v11.9) software (Schrödinger, LLC, New York, NY, USA).

**Tumor xenografts in nude mice**

The Ethics Committee at Shengjing Hospital, affiliated with China Medical University, granted approval for the experiment, which adhered strictly to ethical guidelines for the handling of nude mice. Athymic BALB/c nude mice aged 4 to 6 weeks were sourced from HFK Bioscience Co., Ltd. in Beijing, China. Subsequently, the transfected cells were diluted in a 106/100 μL PBS solution and administered via injection into the nude mice. Following the established protocol, injections were carried out into the axillary tumor location of each mouse every four days. Tumor volumes were monitored every four days over a duration of 28 days, after which the mice were euthanized. Upon tumor identification, photographs were taken for documentation, along with an analysis of the tumor.

**Statistical analysis**

The estimated IC50 values of EC stem cell subtypes were compared utilizing the Kruskal-Wallis test. Survival times of patients with EC were assessed through Kaplan-Meier curves. All statistical analyses were conducted using GraphPad Prism 8 software (GraphPad, Inc., La Jolla, CA, USA). Statistical tests were carried out as two-tailed assessments using R. A p-value of less than 0.05 was regarded as statistically significant.

**Supplementary Table Information**

Additional file Table S1. Primer sequences for qRT-PCR and primary antibodies used for the detection of protein expression.

Additional file Table S2. The 143 genes in the turquoise SCE_M module.

Additional file Table S3. Based on the energy score of the data, 185 compounds with stable binding to CENPL target proteins were selected from the top rankings.

Additional file Table S4. Based on the energy score of the data, 100 compounds with stable binding to SP1 lactylation site at K19 target proteins were selected from the top rankings.

**Supplementary Figures Information**


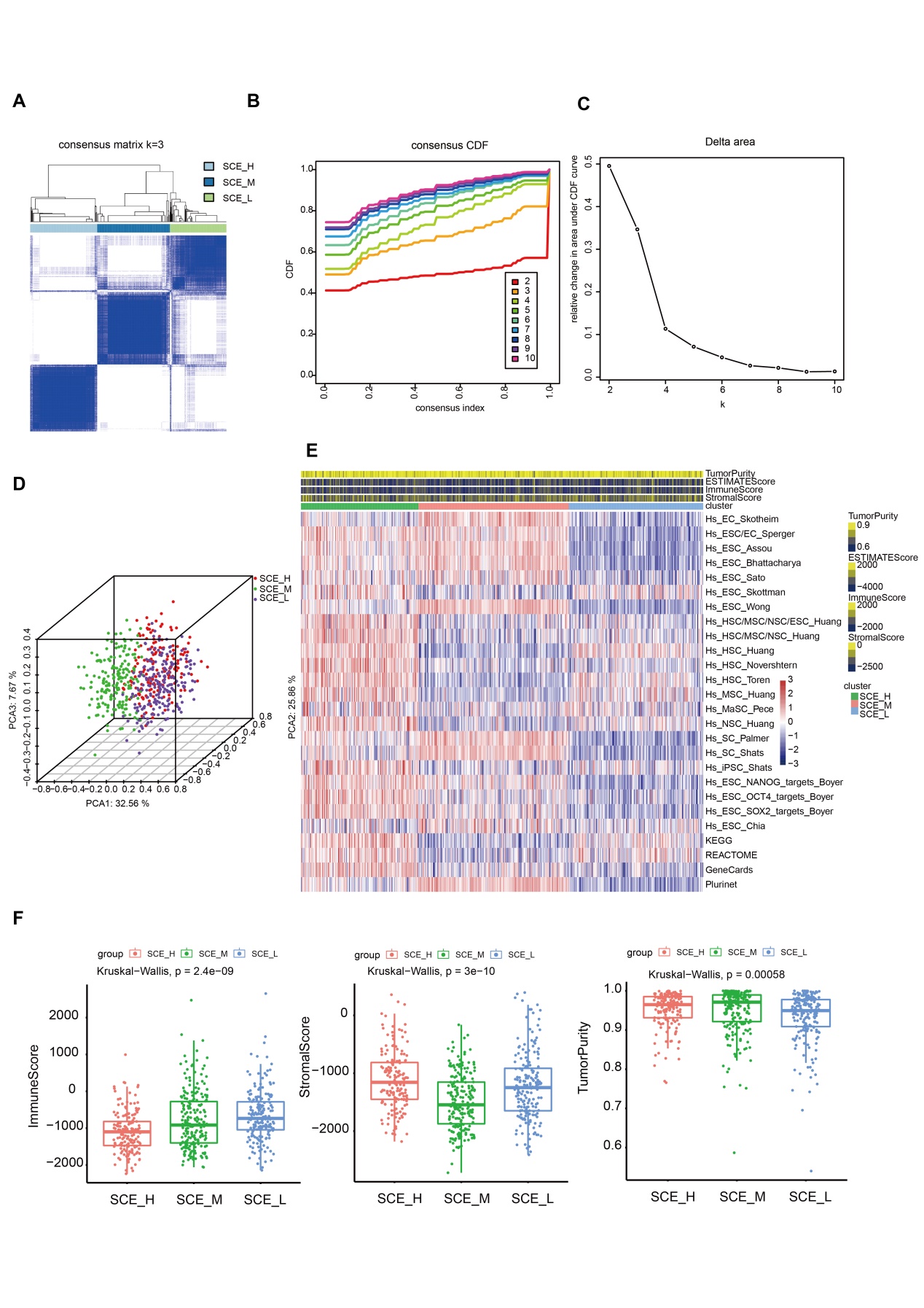


**Figure S1** Consistent clustering of the EC-TCGA cohort. **A** When k = 3, the consensus score matrix of EC samples. The consensus score between the two samples indicates that they are more likely to be assigned to the same cluster in different iterations. **B** Cumulative distribution function (CDF) curve of the consistency score for different subtype numbers (k = 2–10). **C** Delta area plot of the relative increase in cluster stability when k = 3. **D** Principal component analysis (PCA) of samples based on ssGSEA scores, each point represents a sample, and different colours distinguish subtypes. **E** Heatmap of the four stem cell subtypes based on ssGSEA scores for 26 immune gene sets. TCGA, The Cancer Genome Atlas. **F** ESTIMATE was used to evaluate the level of immune infiltration.


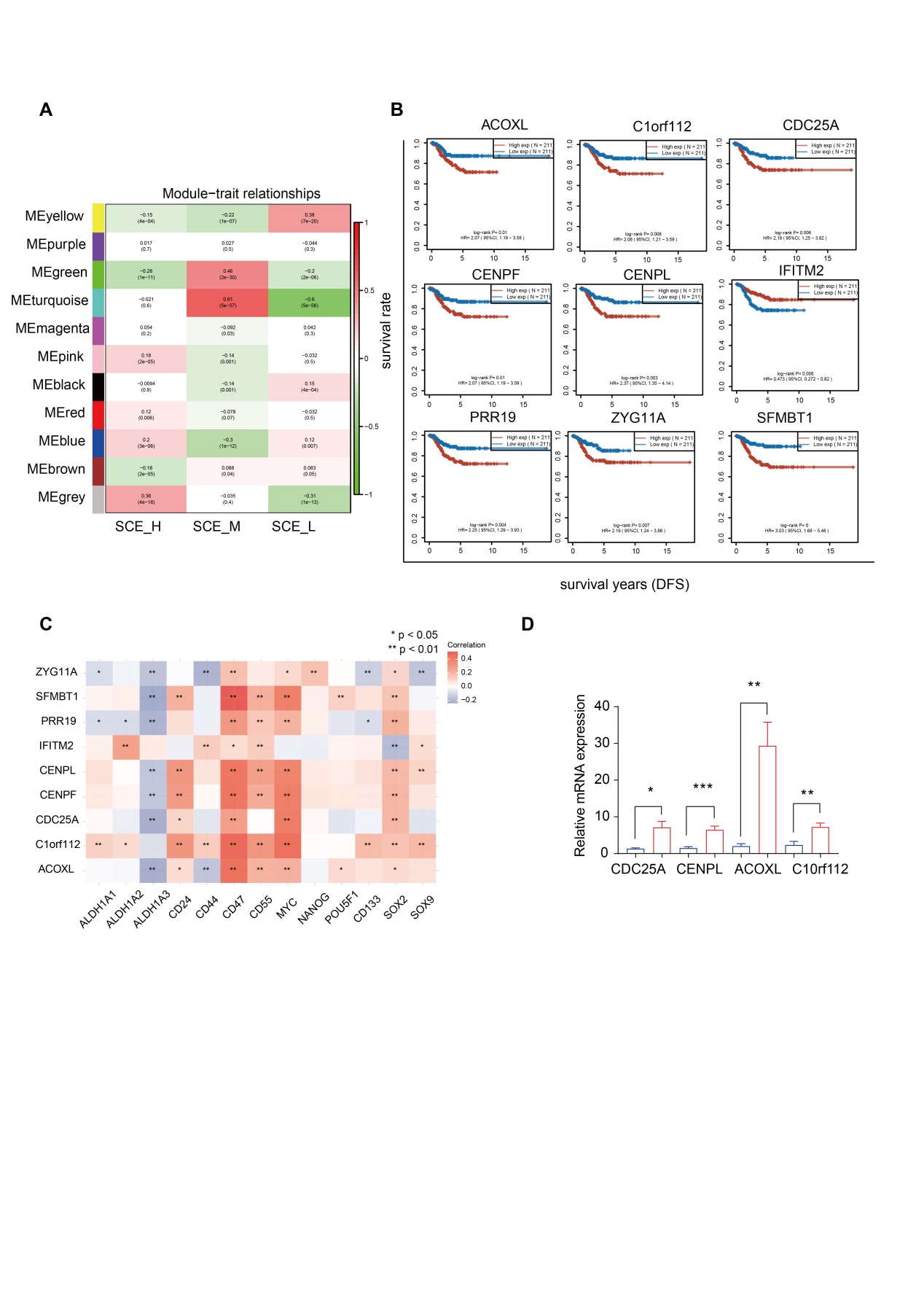


**Figure S2** Screening of prognostic markers associated with the stemness-related genes of endometrial cancer. **A**. Correlation heat map of 11 modules in 3 stem cell subtypes obtained from 3,694 differential genes compared with normal tissues and EC of TCGA cohort. **B** Kaplan–Meier survival curve of nine stemness-related genes prognostic signature. **C** Spearman correlation analysis of the correlation of stemness-related genes with stem cell markers. Red colour represents a positive correlation, blue represents a negative correlation, and the darker the colour, the stronger the correlation; **p* < 0.05, ***p* < 0.01. **D** Expression of the 5 genes in 16 EC and 27 normal tissues, determined using qRT-PCR. **p* < 0.05, ***p* < 0.01, ****p* < 0.001.

**
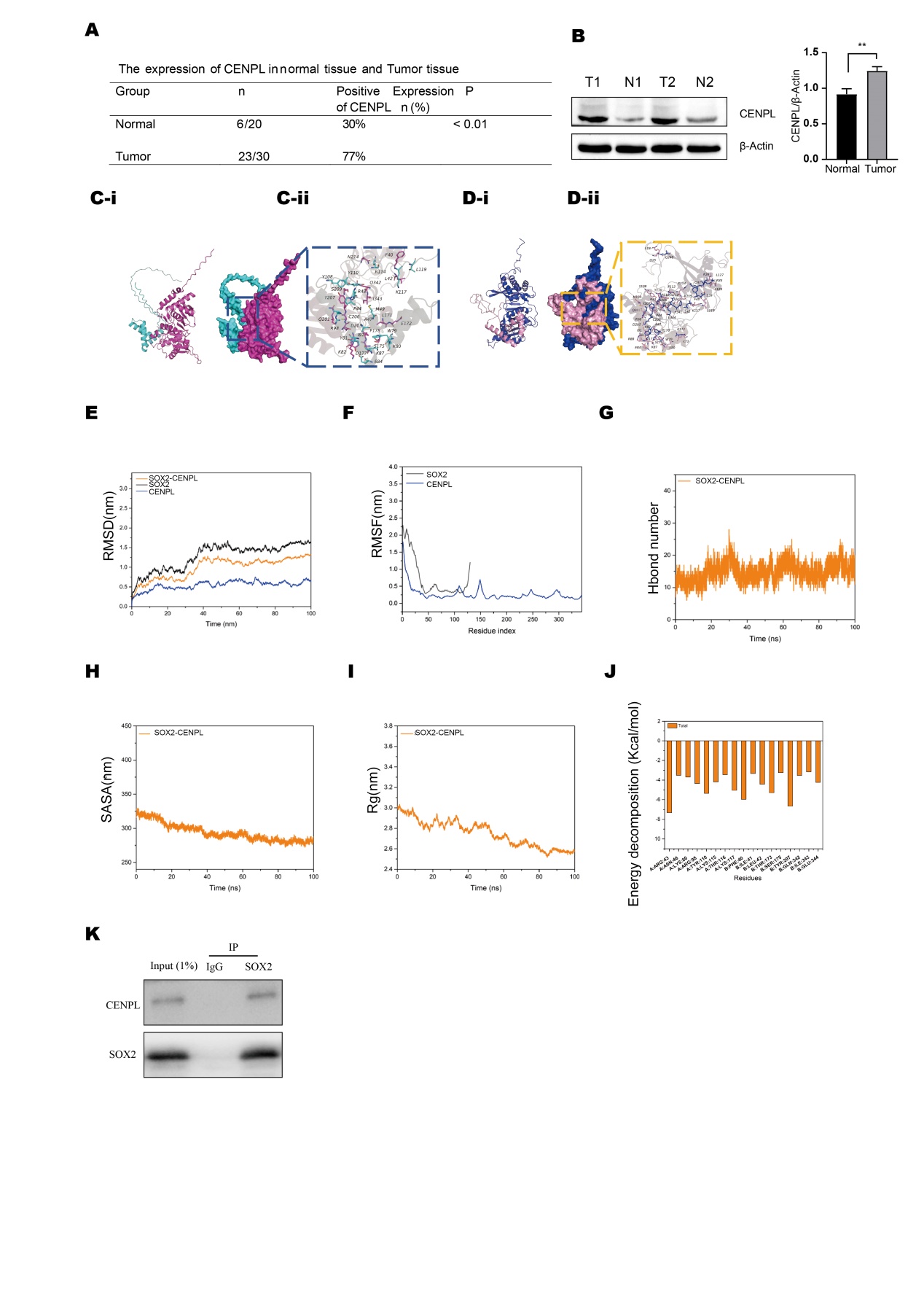
**

**Figure S3** Interaction between SOX2 protein and CENPL. **A** The expression of CENPL in normal endometrial tissue (n=20) and endometrial carcinoma tissue(n=30). **B** Protein expression of CENPL in 20 EC tissues and 12 normal tissues was determined by western blot analysis, **p* < 0.05, ***p* < 0.01. **C** The binding mode of the complex SOX2 with CENPL. **C-i** The backbone of protein was rendered in tube and colored in cyan (SOX2), cyan (VH) and red (VL). SOX2 and CENPL protein is rendered by the surface. **C-ii** The detail binding mode of SOX2 with CENPL. **D** The binding mode of the complex SOX2 with CENPL after 100ns MD simulation. **D**-i The backbone of protein was rendered in tube and colored in pink (SOX2) and blue (CENPL). SOX2 and CENPL protein is rendered by the surface. **D**-ii The detail binding mode of SOX2 with CENPL. **E** RMSD plot during molecular dynamics simulations for protein with protein complex. The wider the distribution range of RMSD values, the richer the changes in molecular conformation. The average RMSD of the complex is less than 2 nm, and the complex reaches dynamic equilibrium at around 50 ns. **F** RMSF plot during molecular dynamics simulations for protein complex. The larger the RMSF value, the greater the conformational fluctuation of amino acids, and the more flexible the movement of amino acid residues. **G** The hydrogen bond number between protein and protein. **H** The surface area changes during the molecular dynamics. SASA，solvent-accessible surface area. The decrease in SASA is mainly due to the enhanced interaction between proteins, resulting in a reduction in the polar area of the protein surface, indicating that the protein has become more compact. **I** The Rg changes of protein complex during the molecular dynamics. Rg evaluates the density of protein structure. The lower the Rg value, the denser and more stable the protein, while the higher the Rg value, the higher the conformational entropy and disorder of the protein. **J** The energy decomposition for the interaction of protein with protein. The negative value of the binding free energy (Δ Gbinding) highlights the stability of the system. The binding free energy between SOX2 and CENPL protein is relatively low (-195.17 ± 9.33 kcal/mol), and van der Waals interaction also contributes significantly to the binding of the two proteins (-164.83 ± 9.08 kcal/mol), indicating stable contact between the two proteins. Combining energy decomposition, we can see that the ARG: 43, ASN: 46, LYS: 80, ARG: 98, TYR: 110, LYS: 115, THR: 116, and LYS: 117 residues of SOX2 protein make significant contributions to their binding; The PHE: 40, ILE: 41, LEU: 42, THR: 173, SER: 175, TYR: 207, GLN: 342, ILE: 343, GLU: 344 residues of CENPL protein contribute significantly to their binding. SOX2 has a strong affinity with CENPL protein and can form a stable complex structure. **K** Co-IP results showing the interaction between CENPL and SOX2.


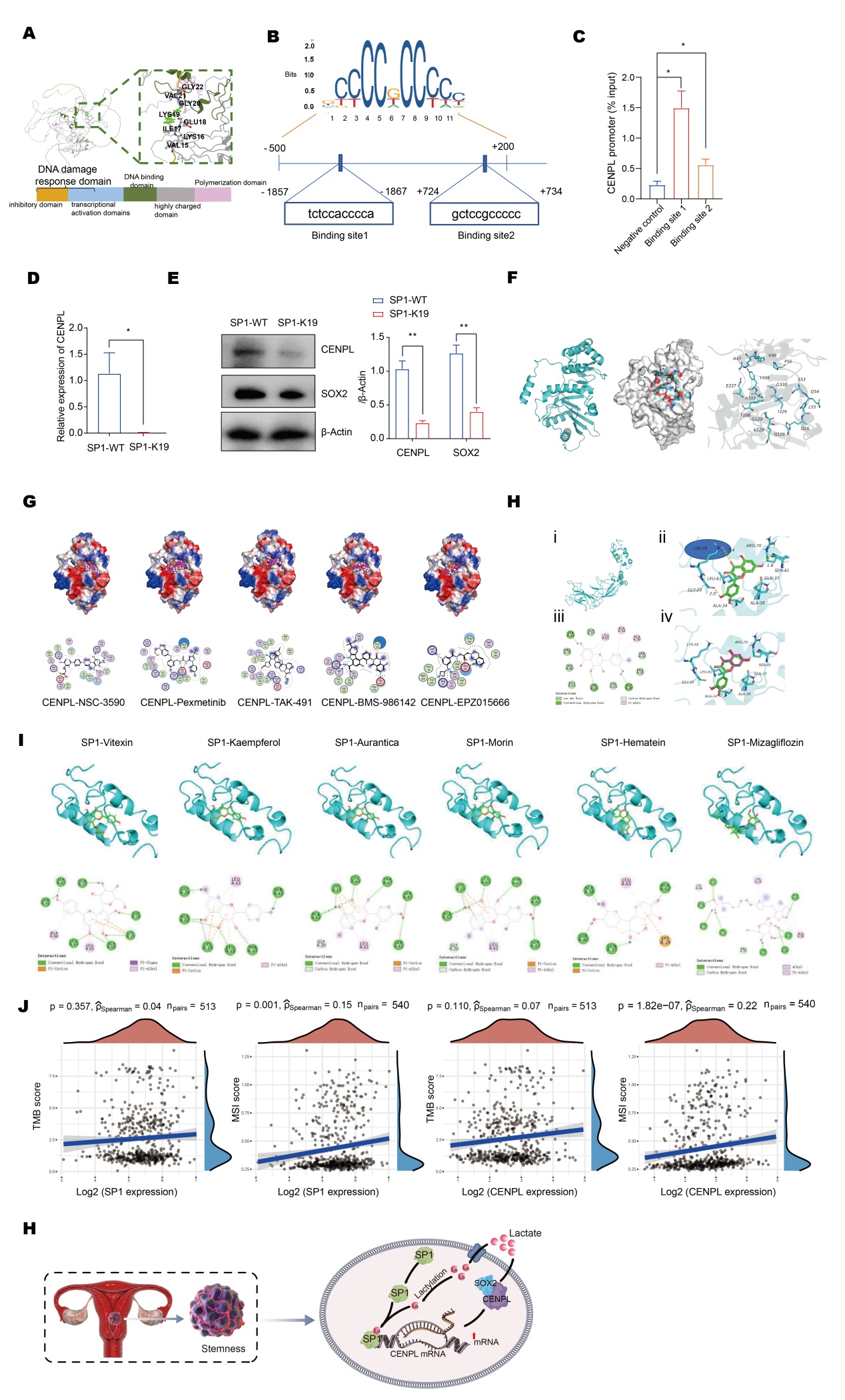


**Figure S4** SP1 lactylation promotes CENPL expression. **A** Ribbon diagram of the human SP1 protein crystal structure. **B**, **C** Potential SP1 binding sites on *CENPL* promoter. Data are presented as mean ± SEM (*n* = 3 per group); **p* < 0.05 compared with the NC group. **D**, **E** CENPL mRNA and protein expression in Ishikawa cells. Data are presented as mean ± SEM (*n* = 3 per group); **p* < 0.05 compared with the WT group. **F** Binding site analysis of crystal structure of CENPL. The 3D structure of CENPL (cyan); binding site surface of CENPL and the important binding site partial of CENPL. **G** Binding mode of CENPL with NSC-3590, Pexmetinib, TAK-491, BMS-986142, EPZ015666. The compounds exhibited stable binding with CENPL target proteins and high matching (binding energy < -6 kcal/mol). **H** The docking model and analysis of SP1. (i) The overall 3D structure of SP1 complex. The backbone of protein was rendered in tube and colored in bright blue and pink. The ref-ligand was rendered in stick and colored by element. (ii) A close view of the active site binding with ref-ligand. Key residues interacted with ref-ligand were rendered in stick and colored by cyan. (iii) The 2D protein-ligand interaction diagram of ligand - SP1 complex. Protein residues were rendered in circle and colored based on their properties: green, hydrophobic residue; purple, polar residue. (iv) The re-docking results of ref-ligand with SP1 target. The ligand molecule can form strong hydrogen bond interactions with key residues LYS-19, SER-41, and GLY-20, with hydrogen bond distances less than 3.5Å. Redocked the Quercetin ligand onto the SP1 binding site. The binding pose overlapped well with the previous complex, with an RMSD<0.7Å. **I** Binding mode of lactylation site at K19 with Vitexin, Kaempferol, Aurantica, Morin, Hematein, Mizagliflozin. The compounds exhibited stable binding with SP1 lactylation site target proteins and high matching (binding energy < -5 kcal/mol). **J** The Spearman correlation analysis between TMB/MSI and SP1, CENPL gene expression. The x-axis represents the distribution of gene expression, and the y-axis represents the score distribution of TMB/MSI. The density curve on the right depicts the distribution trend of TMB/MSI scores, while the density curve on the top shows the distribution trend of gene expression. **H** A working model.
