# Supplementary material for: SP1 lactylation promotes endometrial cancer cell stemness via regulation of CENPL expression
Source: Genes Dis. 2025 Dec 5;13(4):101962. doi: 10.1016/j.gendis.2025.101962 (PMC12989826; doi:10.1016/j.gendis.2025.101962)
Supplement: Multimedia component 2 [file mmc2.docx]

Supplementary file 1: Table S1

Table S1: Primer sequences for qRT-PCR and Primary antibodies used for the detection of protein expression.

| Name | Sequence |
| --- | --- |
| CDC25A | F: GGAAGTACAAAGAGGAGGAAGAG  R: GGGAAGATGCCAGGGATAAA |
| CENPL | F: CTTCCACCTTAGCCTTCCAAGTAGC  R: AATACACAGCAGAACCAGCCAGTC |
| ACOXL  C1orf112 | F: GTGGTGCTATCAGGAATCTCGGAAG  R: GGTCAAAGGTGGCTTCGGTCTG  F: AATTCTGCCCAACCTGTCCTGTATG  R: CGCCTCCAAGGTATGTTGTTCTAGC |
| GAPDH  CENPL-promter1  CENPL-promter2  SP1  SOX2  CENPL  lactylation | F: GCACCGTCAAGGCTGAGAAC R: TGGTGAAGACGCCAGTGGA  F: GAGATGGAGTCTCGCTCTGTCGC R: GGCGCCTGTAGTCCCCGCTG  F: ACATTGGAGTACACGTGTCTAG R: GACTTGTTGAGTAGAAGTGCAG  Manufacturer:CST. USA  Dilution ratio: western blotting, 1:1000  Manufacturer: Affinity Biosciences.OH.USA  Dilution ratio: western blotting, 1:500  Immunofluorescence: 1:100  Manufacturer: Affinity Biosciences.OH.USA  Dilution ratio: western blotting, 1:500  Immunofluorescence: 1:100  Manufacturer: Jingjie PTM BioLabs, Hangzhou, China  Dilution ratio: western blotting, 1:500  Immunohistochemistry: 1:200 |
